# Struma Ovarii: Radiological Features and Diagnostic Challenges

**DOI:** 10.7759/cureus.116271

**Published:** 2026-09-15

**Authors:** Carolina Tim-Tim, Teresa Margarida Cunha

**Affiliations:** 1 Radiology, Instituto Português de Oncologia de Lisboa Francisco Gentil, Lisboa, PRT

**Keywords:** computed tomography, magnetic resonance imaging, multimodality imaging, ovarian neoplasms, ovarian teratoma, struma ovarii, ultrasound

## Abstract

Struma ovarii is a rare monodermal variant of ovarian mature teratoma characterized by the predominance of thyroid tissue, accounting for a small fraction of ovarian germ cell tumors. It demonstrates a highly heterogeneous radiological appearance reflecting its complex internal composition. This variability results in overlapping imaging features with other ovarian tumors, often leading to diagnostic uncertainty in preoperative assessment.

This article summarizes the multimodality radiological characteristics of struma ovarii, with emphasis on ultrasound, computed tomography (CT), and magnetic resonance imaging (MRI). Across imaging modalities, struma ovarii typically presents as a complex adnexal tumor with mixed solid and cystic components, heterogeneous internal architecture, and variable enhancement patterns. MRI provides the most distinctive features, including heterogeneous signal intensity on both T1- and T2-weighted sequences, reflecting differing proportions of thyroid tissue, proteinaceous fluid, and hemorrhagic or colloid content.

Awareness of the imaging spectrum of struma ovarii is essential for radiologists to improve diagnostic accuracy and guide appropriate clinical management. However, definitive diagnosis is rarely achievable on imaging alone, and histopathological examination remains the gold standard for diagnosis.

## Introduction and background

Struma ovarii is a rare form of monodermal ovarian teratoma in which thyroid tissue constitutes more than 50% of the tumor, accounting for approximately 2.7% of all ovarian teratomas [1-3]. Although most cases are benign, malignant transformation occurs in a small percentage of patients [4-6]. Papillary thyroid carcinoma is the most common malignant tumor arising within struma ovarii [4-6].

Clinically, struma ovarii is often asymptomatic or presents with nonspecific pelvic symptoms, making diagnosis challenging [7]. Imaging plays a central role in tumor detection and characterization [7,8]. However, preoperative diagnosis remains challenging because of its heterogeneous and frequently nonspecific radiological appearance, which often overlaps with that of other ovarian neoplasms [7,8]. However, there are certain imaging clues that may aid in the radiological diagnosis of this entity, particularly the detection of “struma pearls” on ultrasound and the characteristic “stained-glass” appearance on MRI [7,8].

This pictorial review describes the multimodality radiological features of struma ovarii, with particular emphasis on ultrasound, computed tomography (CT), and magnetic resonance imaging (MRI), and highlights the principal diagnostic challenges encountered in radiological practice.

## Review

Epidemiology and pathogenesis

Ovarian germ cell tumors originate from primitive germ cells of the embryonic gonad and account for approximately 20%-25% of all ovarian neoplasms [1,3]. Struma ovarii is an uncommon monodermal subtype of mature teratoma and represents a small fraction of ovarian germ cell tumors [1-3].

Histologically, struma ovarii is characterized by the predominance of mature thyroid tissue composed of variably sized follicles lined by thyroid epithelium and filled with colloid, closely resembling normal thyroid gland architecture [1,2]. The lesion is believed to arise from preferential differentiation of totipotent germ cells into thyroid tissue, resulting in ectopic thyroid elements within the ovary [1,2].

The variable proportions of thyroid follicles, colloid material, cystic degeneration, hemorrhage, and fibrosis contribute to the heterogeneous pathological composition of these tumors and largely account for their diverse imaging appearances [7,9].

Clinical presentation

Struma ovarii most commonly presents between the fourth and sixth decades of life [7,10]. The clinical presentation is often nonspecific, with many cases being incidentally detected during imaging or surgical evaluation for an adnexal tumor [7,10]. When symptomatic, patients may present with abdominal or pelvic pain, abdominal distension, or a palpable pelvic mass [7,10]. Less frequently, abnormal vaginal bleeding may occur as part of the nonspecific clinical spectrum of ovarian adnexal lesions [10,11]. Struma ovarii is typically unilateral, with some series reporting a tendency toward left-sided predominance [11].

Endocrine manifestations are uncommon [7,10]. Clinical hyperthyroidism occurs in approximately 5-10% of cases and is attributable to hormonally active thyroid tissue within the tumor [11]. Symptoms may include weight loss, heat intolerance, palpitations, and anxiety, although biochemical thyroid dysfunction may be present even in the absence of overt clinical manifestations [7,11].

In a subset of patients, struma ovarii may be associated with pseudo-Meigs syndrome, characterized by ascites and pleural effusion accompanied by elevated CA-125 levels [12]. These findings may closely mimic advanced ovarian malignancy [12]. Importantly, both effusions and CA-125 elevation are reversible following surgical resection of the tumor [12]. The effusions are usually serous, although serosanguineous fluid may occasionally be observed [12]. The underlying mechanism remains uncertain but is thought to involve a combination of peritoneal irritation, lymphatic or venous obstruction, and tumor-related fluid secretion [12].

Ultrasound findings

On ultrasound, struma ovarii most commonly presents as a multiloculated cystic ovarian tumor with internal solid components of variable echogenicity [10,13]. These features are nonspecific and overlap with those of epithelial ovarian tumors and other germ cell neoplasms [8,10].

A more characteristic but not pathognomonic finding is the presence of so-called “struma pearls,” which correspond to well-circumscribed, round echogenic solid areas with smooth contours and internal vascularity [10,13]. These structures are considered suggestive of struma ovarii, particularly when identified within a predominantly cystic adnexal tumor [10,13].

When a solid, vascularized component is detected in the central portion of an otherwise cystic ovarian tumor, particularly in a lesion that might otherwise be classified as a mature cystic teratoma, struma ovarii should be considered in the differential diagnosis [8,10].

Computed tomography findings

On unenhanced CT, struma ovarii typically appears as a multiloculated cystic tumor [9,14]. Internal high-attenuation components may be observed, reflecting proteinaceous or iodine-rich colloid content within thyroid tissue [9,14]. Calcifications may be detected within the tumor [9].

Following intravenous contrast administration, the lesion demonstrates solid components with enhancement, while cystic areas generally remain non-enhancing [8,9,14]. When struma ovarii coexists with elements of a mature cystic teratoma, areas of fat attenuation may be identified within the lesion, corresponding to sebaceous material or other teratomatous components [14,15]. This mixed composition further contributes to its heterogeneous imaging appearance. Figures 1-3 illustrate the radiological features of struma ovarii on ultrasound and CT.

**Figure 1:**
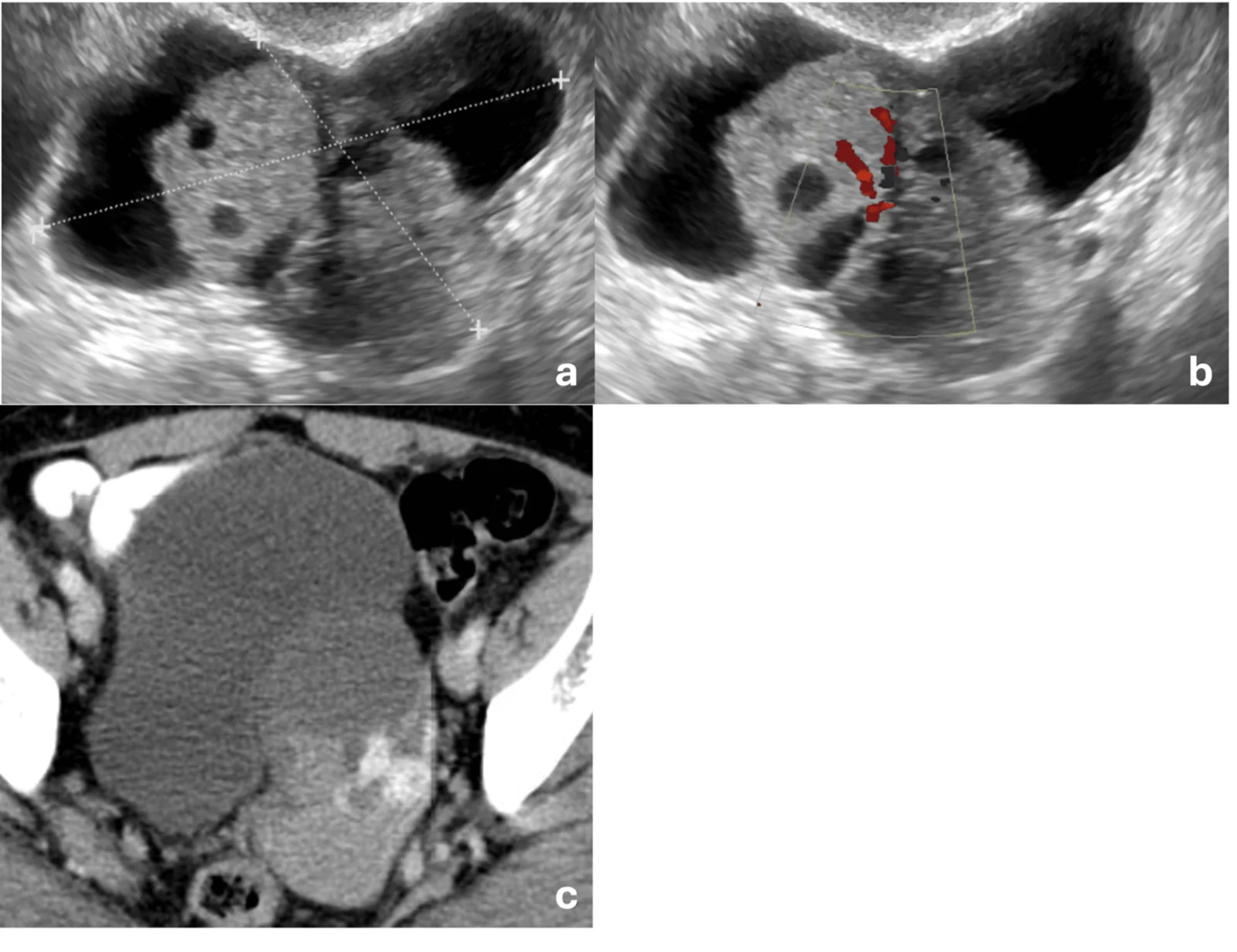
Multicystic ovarian tumor with an enhancing solid component in a patient with struma ovarii Ultrasound of a 56-year-old woman with a large multicystic left ovarian tumor with a central solid component and thick septa on transvaginal ultrasound (a, b). Color Doppler ultrasound demonstrates vascularity within the septa and solid component (b). Axial contrast-enhanced CT image obtained in the venous phase (c) demonstrates a predominantly cystic left ovarian mass with an avidly enhancing solid component. The patient underwent bilateral salpingo-oophorectomy, and histopathological examination of the tumor was consistent with struma ovarii. Image was retrieved from our institution’s database, with appropriate consent for publication obtained.

**Figure 2:**
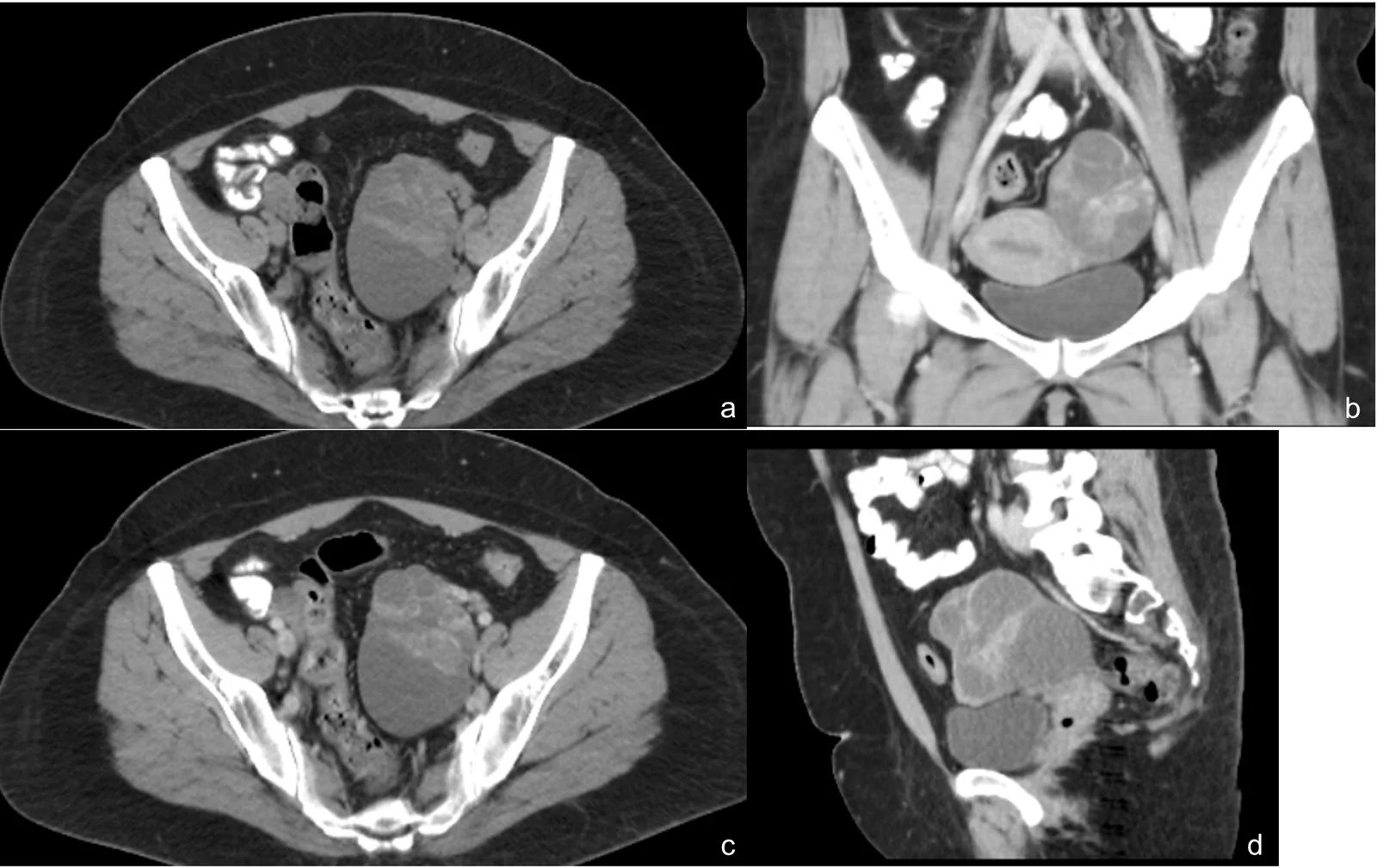
CT appearance of struma ovarii CT of a 56-year-old woman with a multiloculated left ovarian tumor. Axial non-contrast-enhanced CT image (a) and axial contrast-enhanced CT image (c) with coronal (b) and sagittal (d) reconstructions demonstrate enhancing solid areas within the predominantly cystic tumor. The patient underwent bilateral salpingo-oophorectomy, and histopathological examination was consistent with struma ovarii. Image was retrieved from our institution’s database, with appropriate consent for publication obtained.

**Figure 3:**
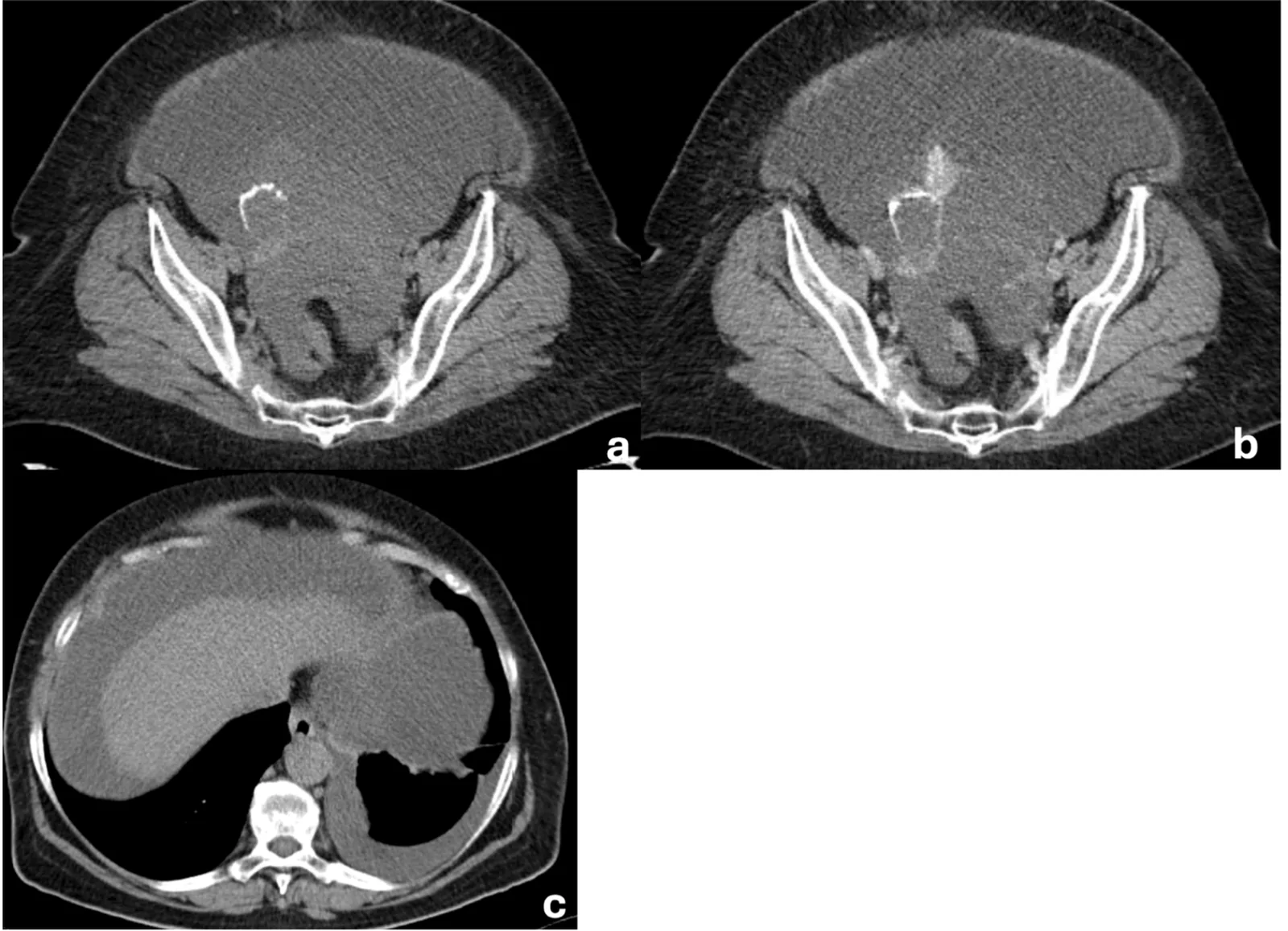
Pseudo-Meigs syndrome associated with struma ovarii CT of a 66-year-old female patient presented with rapid weight gain and abdominal distension. Serum CA-125 levels were elevated, initially raising suspicion of ovarian malignancy. The patient was ultimately diagnosed with pseudo-Meigs syndrome associated with benign struma ovarii. Imaging demonstrated ascites (a and b) and pleural effusion (c). (a) Non-contrast-enhanced CT revealed a pelvic mass with internal calcifications, while (b) contrast-enhanced CT demonstrated avid enhancement of a solid component within the lesion. Image was retrieved from our institution’s database, with appropriate consent for publication obtained.

Additional findings such as ascites and pleural effusion may occasionally be present, particularly in cases associated with pseudo-Meigs syndrome, as illustrated in Figure 3, potentially mimicking malignant ovarian neoplasms [12].

Magnetic resonance findings

On MRI, struma ovarii typically presents as a multiloculated cystic tumor with solid components and thickened septa [14,16]. The cystic locules demonstrate variable signal intensity on both T1- and T2-weighted sequences, reflecting the heterogeneous internal composition of the lesion, including colloid, proteinaceous material, and occasional hemorrhagic content [14,16]. This variability gives rise to the distinctive “stained-glass appearance,” in which multiple locules exhibit differing signal characteristics, ranging from high T1 and relatively low T2 signal intensity to intermediate or fluid-like signal patterns [14,16]. Although not pathognomonic, this so-called “stained-glass appearance” is considered a characteristic MRI feature of struma ovarii and may provide an important diagnostic clue [7,14,16].

Locules demonstrating very low signal intensity on both T1- and T2-weighted sequences are believed to contain highly viscous colloid material [14,16]. The exact mechanism underlying this signal pattern is thought to be related to the physicochemical properties of concentrated colloid [9].

The solid components, often corresponding to thyroid tissue or thickened septa, typically demonstrate enhancement of variable degree following gadolinium administration [8,14,16].

Figures 4-9 illustrate the imaging features of struma ovarii on MRI. Among these, Figures 4, 5 also demonstrate the findings identified on the ultrasound examination performed prior to MRI.

**Figure 4:**
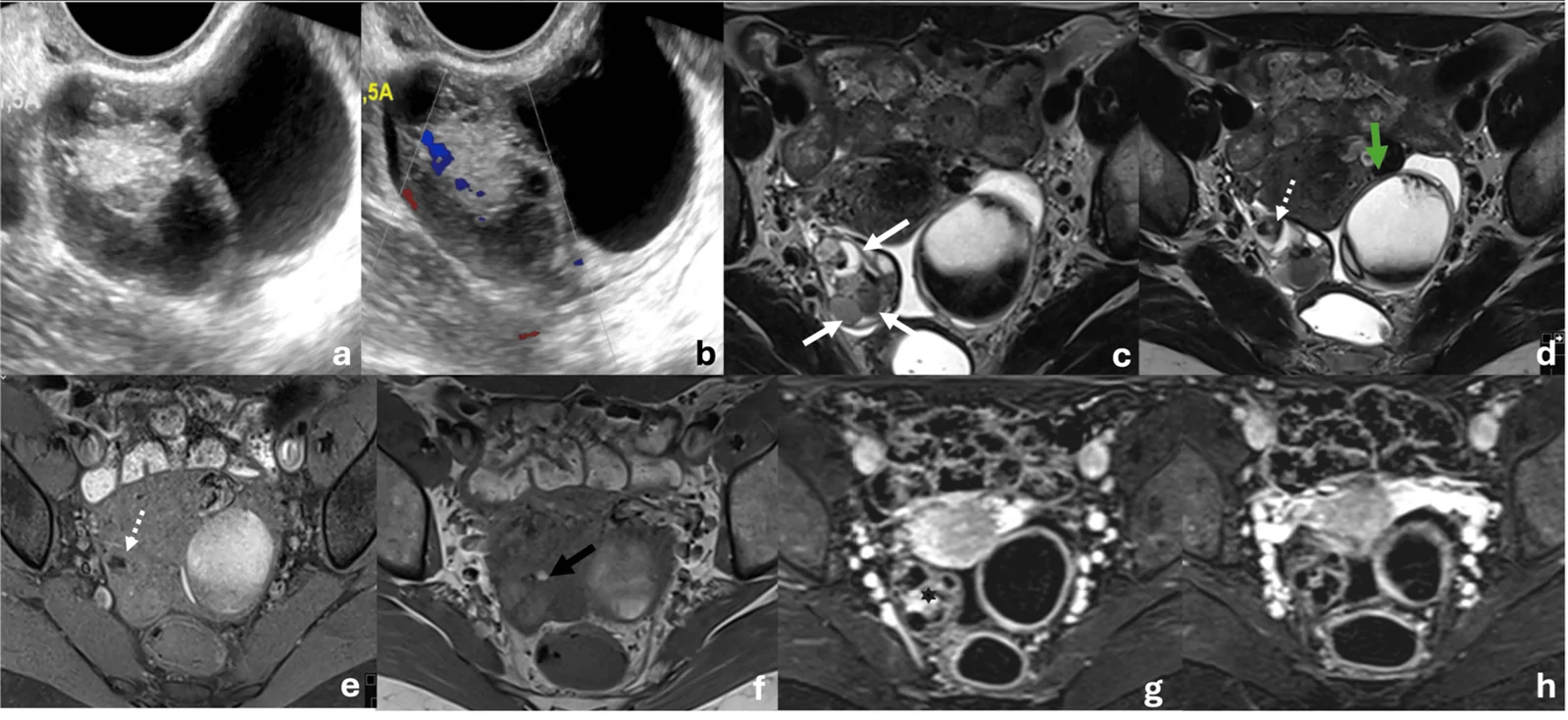
Multimodality imaging of struma ovarii Ultrasound of a 35-year-old woman with a history of prior laparoscopic cystectomy of the left ovary, currently diagnosed with right ovarian struma ovarii. Transvaginal ultrasound images demonstrate a large multiloculated right ovarian mass with echogenic fluid, thick septations, and a central solid component exhibiting internal vascularity on color Doppler evaluation. Axial T2-weighted images (c, d) show a complex multicystic lesion with locules of varying signal intensity (white arrows). A locule demonstrating marked hypointensity on both T1-weighted (e) and T2-weighted (d) images likely corresponds to gelatinous colloid content (dashed arrows). A small anterior locule demonstrates hyperintensity on T1-weighted imaging (f - black arrow). Post-gadolinium subtracted T1-weighted images (g, h) demonstrate avid enhancement of a solid component (asterisk) within a predominantly non-enhancing lesion. A hemorrhagic corpus luteum in the contralateral ovary is also noted on axial T2-weighted images (c, d - green arrow). Image was retrieved from our institution’s database, with appropriate consent for publication obtained.

**Figure 5:**
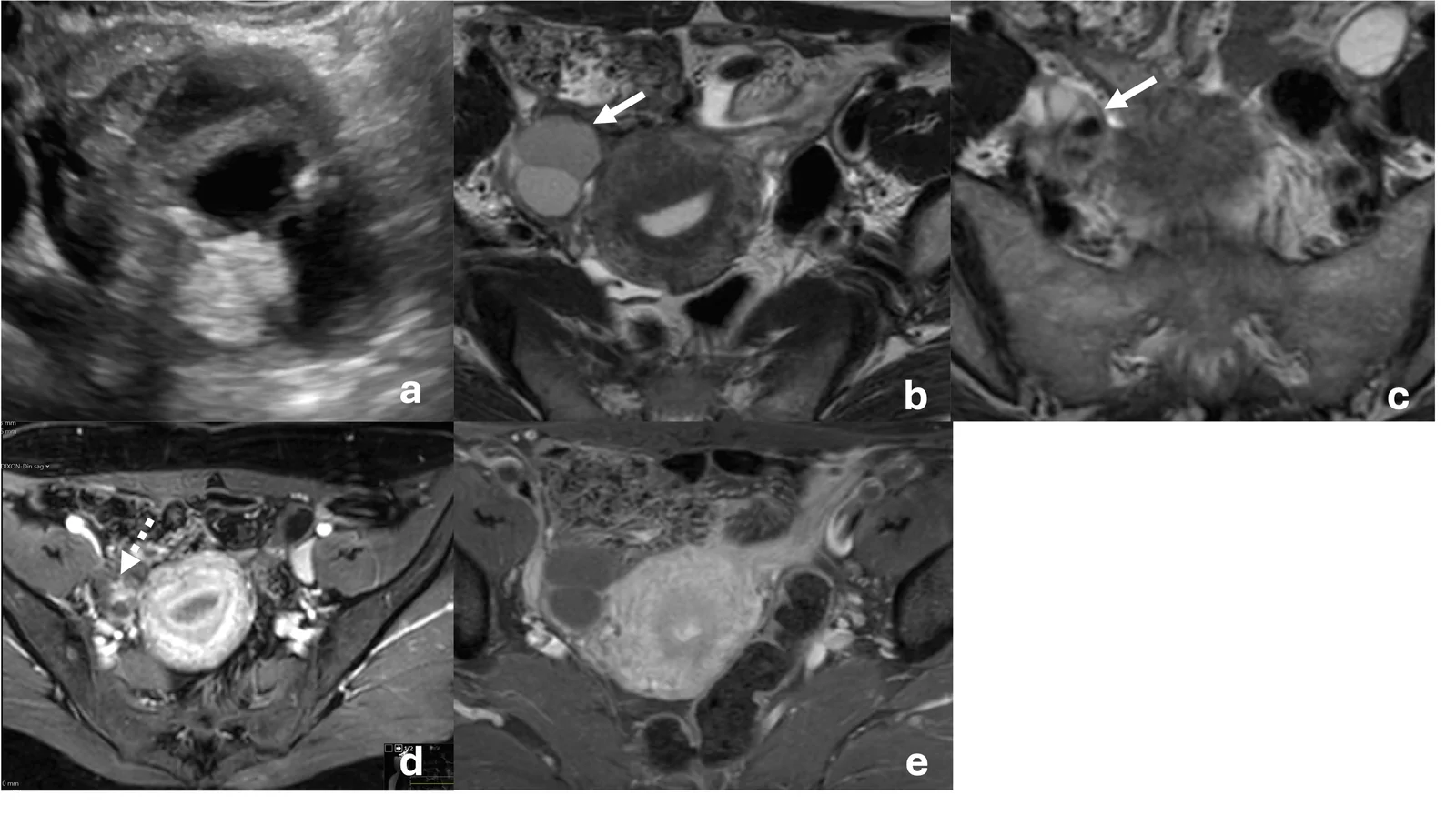
Multicystic ovarian tumor with a “stained-glass” appearance on MRI MRI of a 46-year-old woman with a right ovarian multiloculated cystic tumor. Sagittal T2-weighted magnetic resonance image (a) and axial T2-weighted images (b, c) demonstrate a multiloculated cystic mass with locules of variable signal intensity, producing a characteristic “ground-glass” appearance. A locule with marked low signal intensity on T2-weighted imaging (white arrow, c) and corresponding marked low signal intensity on T1-weighted imaging (white arrow, d) likely reflects thick colloid. Axial T1-weighted fat-suppressed magnetic resonance image obtained after gadolinium administration (e) demonstrates enhancing solid areas within the lesion. The patient underwent surgical excision, and histopathologic examination was consistent with struma ovarii. Image was retrieved from our institution’s database, with appropriate consent for publication obtained.

**Figure 6:**
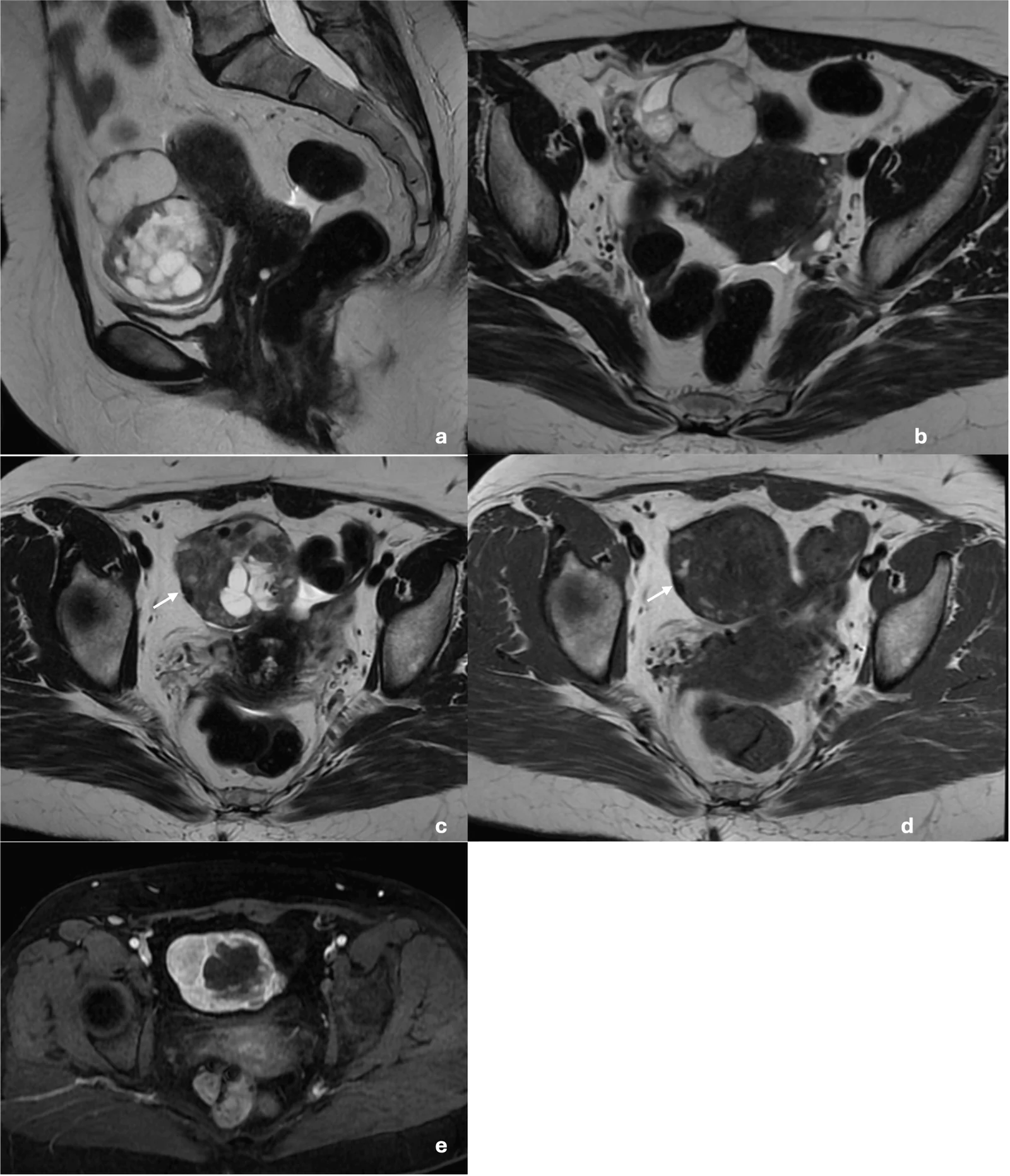
Multiloculated ovarian tumor with variable signal intensity and enhancing solid components in struma ovarii MRI of a 46-year-old woman with a right ovarian multiloculated cystic tumor. Sagittal T2-weighted magnetic resonance image (a) and axial T2-weighted images (b, c) demonstrate a multiloculated cystic mass with locules of variable signal intensity, producing a characteristic “ground-glass” appearance. A locule with marked low signal intensity on T2-weighted imaging (white arrow, c) and corresponding marked low signal intensity on T1-weighted imaging (white arrow, d) likely reflects thick colloid. Axial T1-weighted fat-suppressed magnetic resonance image obtained after gadolinium administration (e) demonstrates enhancing solid areas within the lesion. The patient underwent surgical excision, and histopathologic examination was consistent with struma ovarii. (Image retrieved from our institution’s database, with appropriate consent for publication obtained).

**Figure 7:**
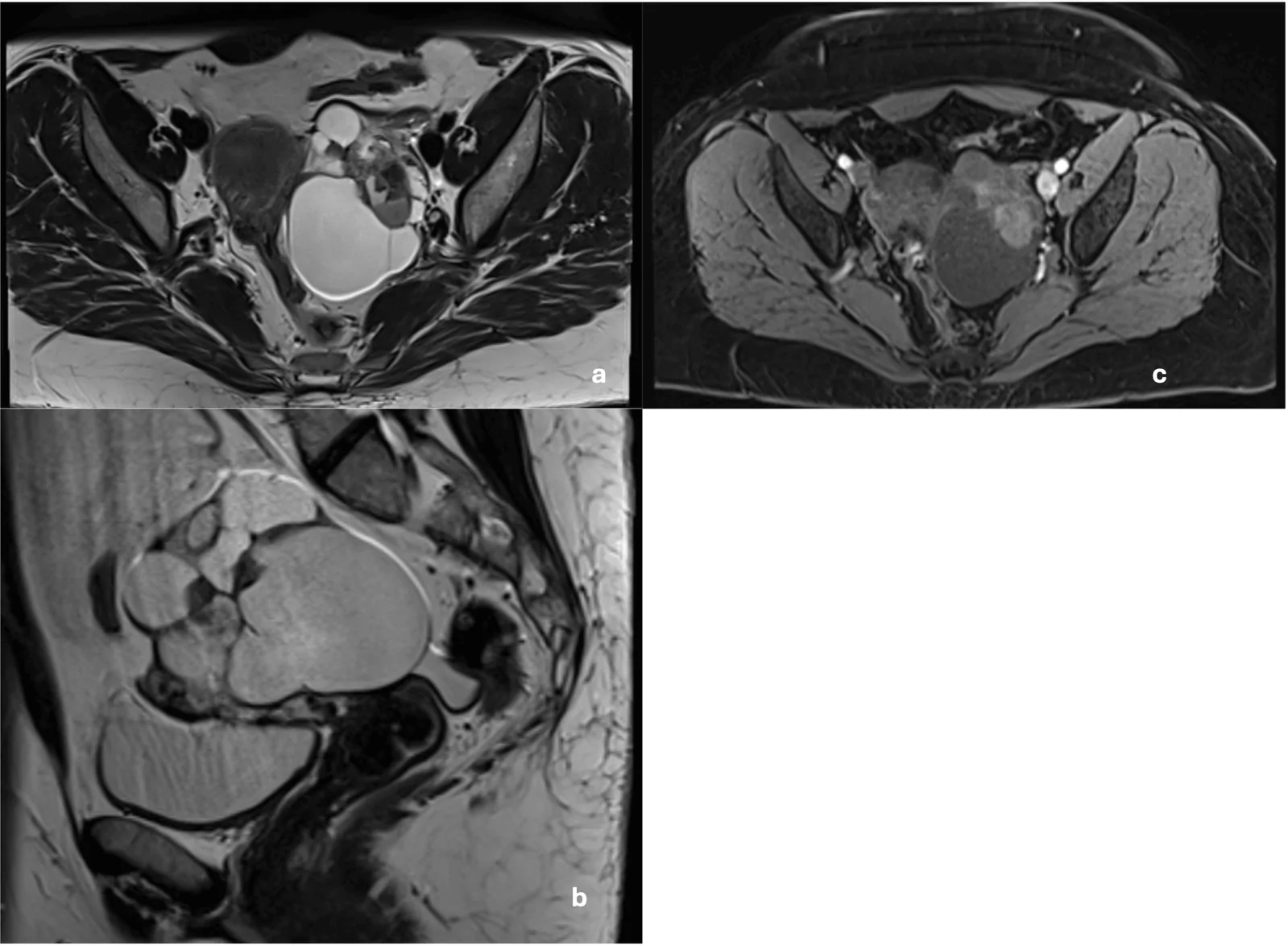
Multiloculated cystic ovarian tumor with imaging features suspicious for malignancy MRI of a 40-year-old woman with a multiloculated cystic tumor arising from the left ovary. Axial and sagittal T2-weighted magnetic resonance images (a and b, respectively) demonstrate a multiloculated cystic mass with locules of variable signal intensity, producing a characteristic “ground-glass” appearance. Axial T1-weighted fat-suppressed magnetic resonance image obtained after gadolinium administration (c) demonstrates thick enhancing septa and solid vegetations, findings that raise suspicion for malignancy. The patient underwent surgical excision, and histopathologic examination was consistent with benign struma ovarii. Image was retrieved from our institution’s database, with appropriate consent for publication obtained.

**Figure 8:**
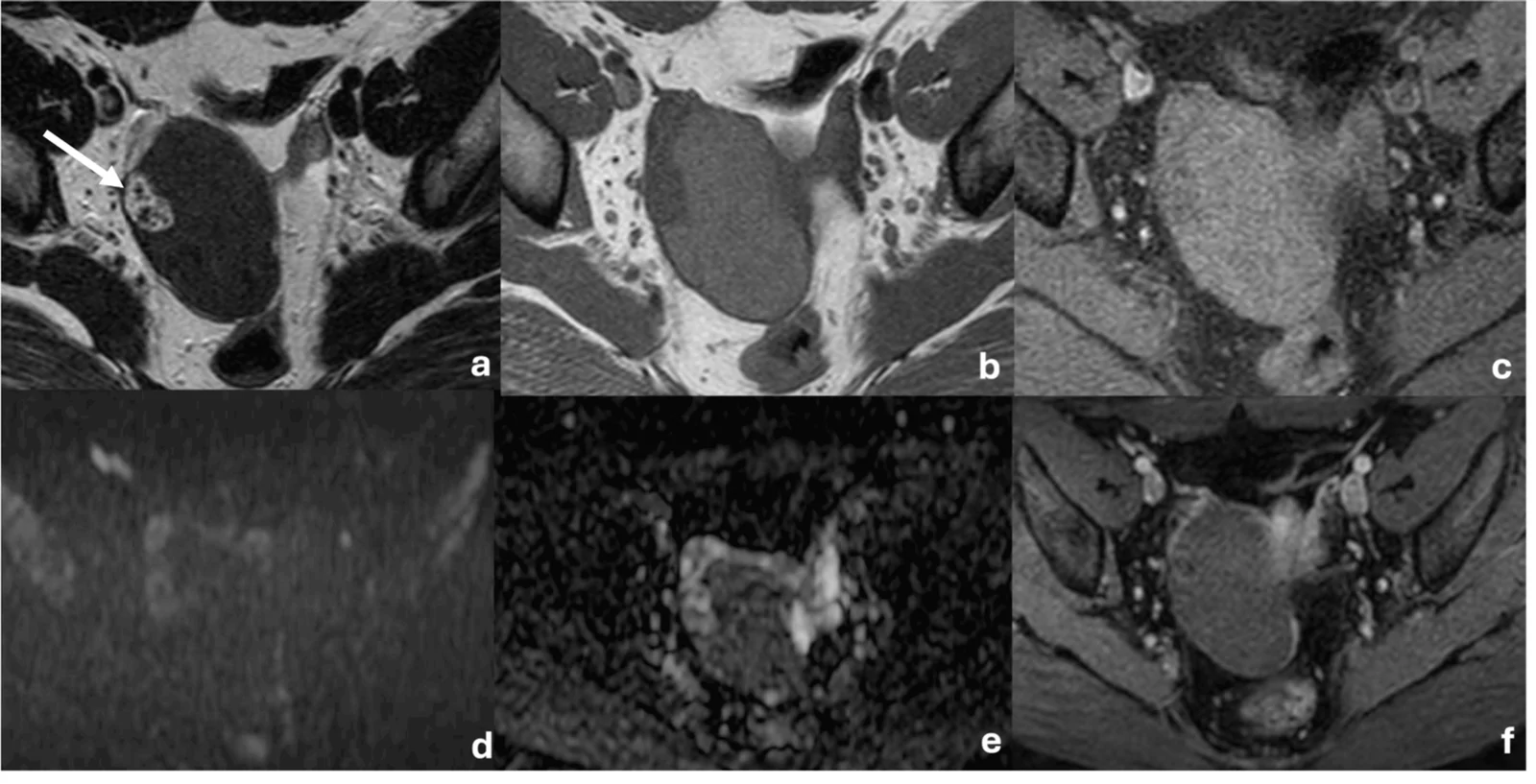
MRI appearance of struma ovarii MRI of a 16-year-old female patient with histologically confirmed struma ovarii. MRI demonstrates a 6-cm oval-shaped cystic tumor in the right ovary, showing low signal intensity on T2-weighted imaging (a) and intermediate signal intensity on T1-weighted and fat-suppressed T1-weighted images (b, c). An eccentric mural nodule (white arrow) demonstrates high signal intensity on T2-weighted imaging. No macroscopic fat is identified. The lesion does not demonstrate restricted diffusion (d, e) and no enhancement following gadolinium administration (f). Image was retrieved from our institution’s database, with appropriate consent for publication obtained.

**Figure 9:**
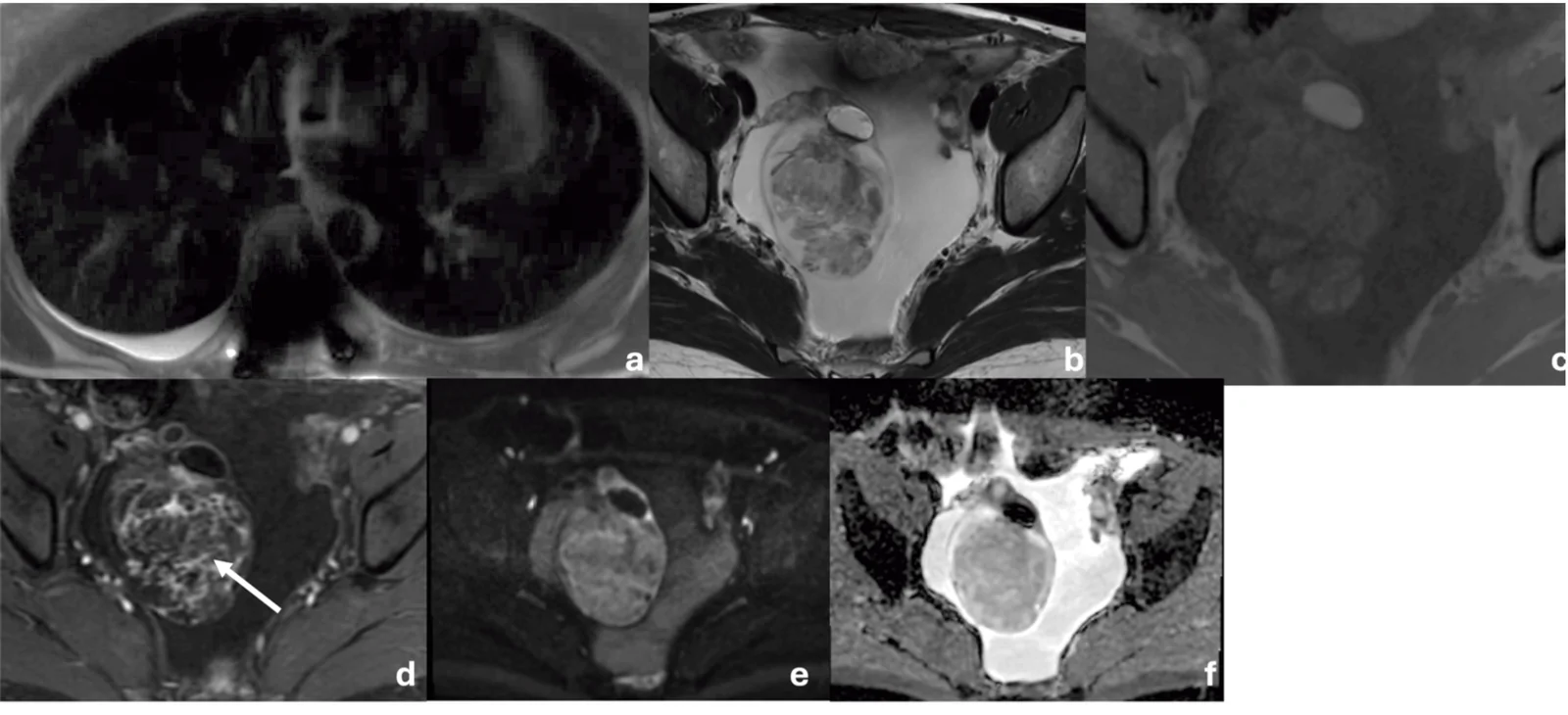
Pseudo-Meigs syndrome associated with benign struma ovarii MRI of a 38-year-old woman with pseudo-Meigs syndrome in the setting of benign struma ovarii. Pleural effusion and ascites are demonstrated (a, b). Axial T2-weighted imaging (b) shows a large multicystic right ovarian tumor with areas of intermediate signal intensity. Axial T1-weighted imaging (c) demonstrates locules with high signal intensity. Post-gadolinium subtracted T1-weighted imaging (d) shows a smooth, web-like (“lacy”) pattern of enhancement corresponding to the internal septations (white arrow). Diffusion-weighted imaging (e) and the corresponding apparent diffusion coefficient (ADC) map (f) demonstrate no evidence of restricted diffusion. Image was retrieved from our institution’s database, with appropriate consent for publication obtained.

As described in the CT findings, ascites and pleural effusion may also occasionally be identified on MRI in the setting of pseudo-Meigs syndrome associated with struma ovarii, as illustrated in Figure 9.

Although MRI is considered the most informative modality for characterizing struma ovarii, its imaging features remain nonspecific and may overlap with those of other complex ovarian cystic neoplasms, particularly epithelial ovarian tumors and cystic teratomas [7,8,14,16].

Table 1 summarizes the radiological findings of struma ovarii across multiple imaging modalities.

**Table 1:** Radiological Findings in Struma Ovarii Summary of the characteristic imaging findings of struma ovarii on ultrasound, CT, and MRI. CT, computed tomography; MRI, magnetic resonance imaging

Modality	Key Radiological Findings
Ultrasound	Multiloculated cystic ovarian tumor with internal solid components of variable echogenicity (nonspecific; overlaps with epithelial ovarian tumors and other germ cell neoplasms). Characteristic finding: "struma pearls" - well-circumscribed, round, echogenic solid areas with smooth contours and internal vascularity, particularly within a predominantly cystic adnexal tumor.
CT	Unenhanced CT: multiloculated cystic tumor with internal high-attenuation components reflecting proteinaceous/iodine-rich colloid content; calcifications may be present. Post-contrast: enhancing solid components with non-enhancing cystic areas. Fat-attenuation foci may be seen when coexisting with a mature cystic teratoma.
MRI	Multiloculated cystic tumor with solid components and thickened septa. Characteristic "stained-glass appearance": variable signal intensity across locules on T1- and T2-weighted sequences (high T1/low T2 to intermediate or fluid-like signal), reflecting heterogeneous content (colloid, proteinaceous material, occasional hemorrhage). Locules with very low T1 and T2 signal intensity suggest highly viscous colloid. Solid components/septa show variable enhancement after gadolinium administration.

Malignant transformation

Although the vast majority of cases are benign, malignant transformation has been reported in approximately 5% of struma ovarii lesions [4-6]. The most common malignant subtype is papillary thyroid carcinoma arising within the ovarian thyroid tissue, accounting for approximately 70% of malignant cases, with the classical and follicular variants representing approximately 44% and 26%, respectively [6].

Malignant transformation has been reported among large tumor sizes, with most malignant cases occurring in lesions measuring more than 16 cm, whereas malignant tumors smaller than 5 cm are rare [17]. Metastatic disease is uncommon, and when present, dissemination most frequently involves the adjacent pelvic structures. Distant metastatic spread, involving organs such as the lungs, bones, liver, skin, or brain, is exceedingly uncommon [17].

From a diagnostic imaging perspective, no specific radiological features reliably distinguish benign from malignant struma ovarii (Figure 10) in the absence of metastatic disease or overt invasive behavior, such as capsular or extra-ovarian extension [4,5].

**Figure 10:**
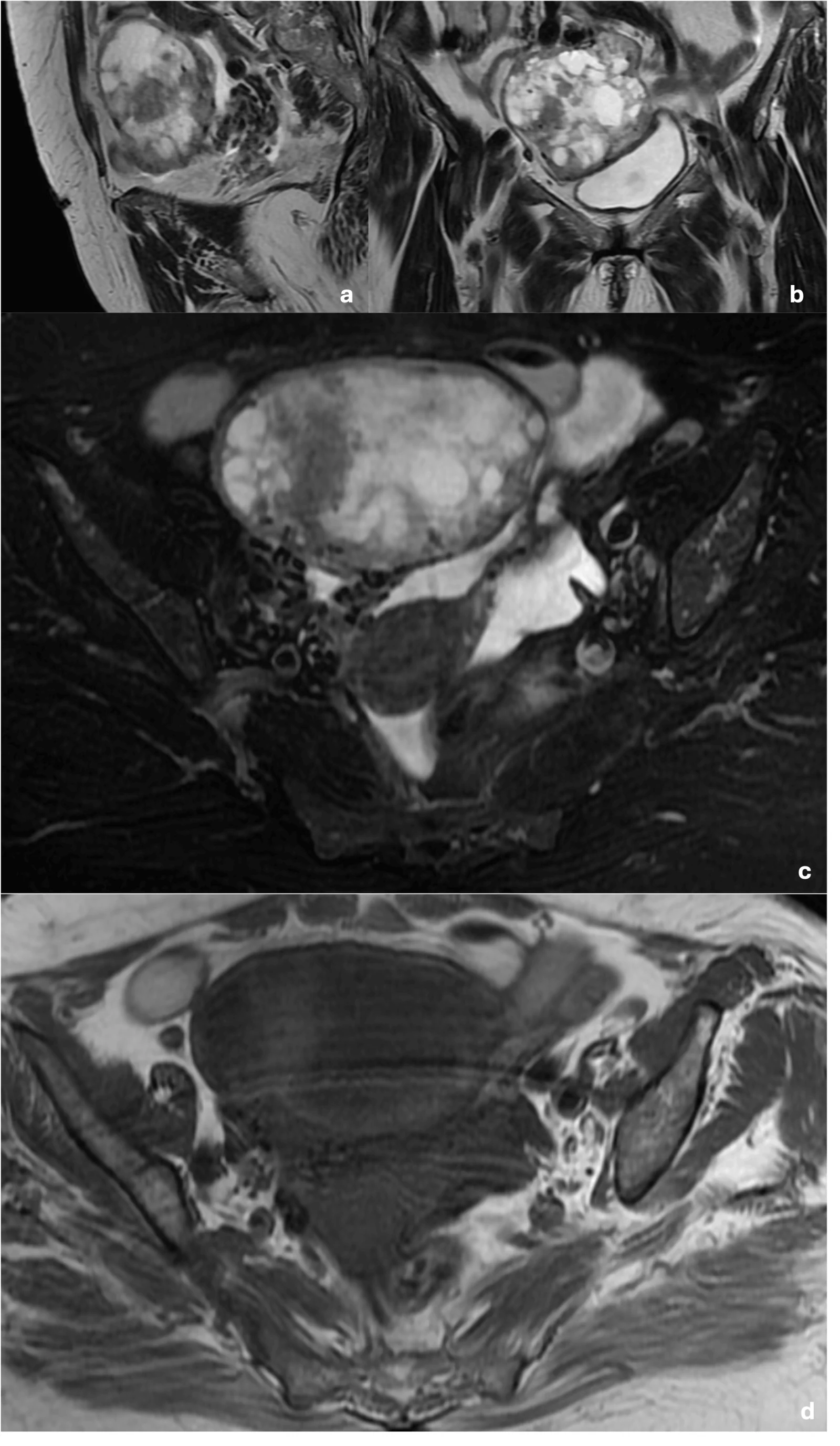
Papillary carcinoma arising in struma ovarii MRI of a 71-year-old woman presenting with an ovarian lesion histologically demonstrating thyroid follicles and foci of papillary carcinoma, consistent with papillary carcinoma arising in struma ovarii. Sagittal and coronal T2-weighted (a, b), axial T2-weighted fat-suppressed (c), and axial T1-weighted (d) images demonstrate a multiloculated cystic mass with locules of variable signal intensity and thick septa. Some locules show low signal intensity on T1-weighted images and high signal intensity on T2-weighted images, whereas others demonstrate intermediate signal intensity on both T1-weighted and T2-weighted images. No macroscopic fat is identified. A small amount of ascites is present in the pouch of Douglas. Image was retrieved from our institution’s database, with appropriate consent for publication obtained.

Differential diagnosis

The imaging appearance of struma ovarii overlaps with a range of ovarian neoplasms [7,8]. Mature cystic teratoma and mucinous neoplasms represent the most important differential diagnoses based on radiological features [14,18].

Mature cystic teratomas may closely resemble struma ovarii on ultrasound, particularly when presenting as a cystic lesion with an echogenic mural nodule (Rokitansky nodule) [13,15]. However, in contrast to struma ovarii, vascular flow is typically absent within the nodule, with Doppler signal usually confined to the peripheral capsule [13,15]. The presence of internal vascularity within a solid component should raise suspicion for an alternative diagnosis, specifically struma ovarii [13]. Teratomas may also present as diffusely echogenic masses due to sebaceous material or hair or as cystic lesions containing multiple linear echogenic strands [15]. Calcifications, including teeth or wall calcifications, may be present and are best demonstrated on CT [15]. Fat attenuation on CT is highly sensitive for the diagnosis of mature cystic teratoma and may occasionally present as a fat-fluid level [15]. On MRI, intralesional fat typically demonstrates high signal intensity on T1-weighted images with signal loss on fat-suppressed sequences [16]. Chemical shift imaging may further aid diagnosis in cases without macroscopic fat by demonstrating signal drop on out-of-phase sequences [16].

Mucinous ovarian tumors, including cystadenomas and carcinomas, may be difficult to distinguish from struma ovarii due to their shared multiloculated cystic architecture and variable signal intensity on CT and MRI [18]. The “stained-glass appearance” may also be present in mucinous tumors, reflecting variable mucin concentration, with thick mucin demonstrating high T1 and low T2 signal intensity, whereas watery mucin shows the opposite pattern [18]. Distinction between benign and malignant mucinous tumors relies on morphological features [18]. Carcinomas typically demonstrate solid components, as well as thick or irregular septa and wall nodularity, whereas cystadenomas usually present with thin, smooth septations and walls [18]. Calcifications may also be present in mucinous tumors [19].

Importantly, struma ovarii is frequently associated with other ovarian tumor components, with approximately 50%-60% of cases coexisting with mature cystic teratomas and, less commonly, with mucinous cystadenomas or carcinoid tumors [2]. This association further contributes to its heterogeneous imaging appearance and diagnostic complexity.

Despite these overlapping radiological features, ancillary findings may suggest the diagnosis of struma ovarii, including “struma pearls” on ultrasound [10,13], high-attenuation locules on CT related to proteinaceous and iodine-rich colloid content [9,14], and locules demonstrating very low signal intensity on both T1- and T2-weighted sequences related to highly viscous colloid content [14,16]. Nevertheless, imaging findings remain nonspecific [4,7].

Metastatic thyroid carcinoma to the ovary is an exceptionally rare entity and represents a critical diagnostic pitfall [5]. Reported cases are almost exclusively associated with a known history of primary papillary thyroid carcinoma with disseminated disease [5]. For this diagnosis to be established, no benign struma ovarii or teratomatous components should be identified on histopathological evaluation [5].

## Conclusions

Struma ovarii is a rare ovarian tumor with variable and often nonspecific radiological features that frequently overlap with those of other ovarian neoplasms. Although certain imaging patterns, such as the “stained-glass” appearance on MRI, “struma pearls” on ultrasound, and high-attenuation locules on CT, may suggest the diagnosis, none are pathognomonic.

Multimodality imaging can provide important diagnostic clues, substantially narrow the differential diagnosis, and guide appropriate clinical management. However, definitive diagnosis remains based on histopathological evaluation.

Knowledge of the characteristic imaging spectrum of struma ovarii is essential for radiologists to improve diagnostic confidence and ensure accurate preoperative assessment, ultimately contributing to optimal patient management.

## References

[1] Talerman A (2002). Germ cell tumors of the ovary. Blaustein’s Pathology of the Female Genital Tract. 5th ed.

[2] Roth LM, Talerman A (2007). The enigma of struma ovarii. Pathology.

[3] Shaaban AM, Rezvani M, Elsayes KM (2014). Ovarian malignant germ cell tumors: cellular classification and clinical and imaging features. Radiographics.

[4] Kostoglou-Athanassiou I, Lekka-Katsouli I, Gogou L, Papagrigoriou L, Chatonides I, Kaldrymides P (2002). Malignant struma ovarii: report of a case and review of the literature. Horm Res.

[5] Leite I, Cunha TM, Figueiredo JP, Félix A (2013). Papillary carcinoma arising in struma ovarii versus ovarian metastasis from primary thyroid carcinoma: a case report and review of the literature. J Radiol Case Rep.

[6] Salman WD, Singh M, Twaij Z (2010). A case of papillary thyroid carcinoma in struma ovarii and review of the literature. Patholog Res Int.

[7] Dujardin MI, Sekhri P, Turnbull LW (2014). Struma ovarii: role of imaging?. Insights Imaging.

[8] Taylor EC, Irshaid L, Mathur M (2021). Multimodality imaging approach to ovarian neoplasms with pathologic correlation. Radiographics.

[9] Ikeuchi T, Koyama T, Tamai K, Fujimoto K, Mikami Y, Konishi I, Togashi K (2012). CT and MR features of struma ovarii. Abdom Imaging.

[10] Savelli L, Testa AC, Timmerman D, Paladini D, Ljungberg O, Valentin L (2008). Imaging of gynecological disease (4): clinical and ultrasound characteristics of struma ovarii. Ultrasound Obstet Gynecol.

[11] Tomee JF, van der Heijden PF, van den Hout JH, Brinkhuis M, Veneman TF (2008). Papillary carcinoma in struma ovarii: an unusual presentation. Neth J Med.

[12] Jiang W, Lu X, Zhu ZL, Liu XS, Xu CJ (2010). Struma ovarii associated with pseudo-Meigs' syndrome and elevated serum CA 125: a case report and review of the literature. J Ovarian Res.

[13] Zalel Y, Caspi B, Tepper R (1997). Doppler flow characteristics of dermoid cysts: unique appearance of struma ovarii. J Ultrasound Med.

[14] Shen J, Xia X, Lin Y, Zhu W, Yuan J (2011). Diagnosis of Struma ovarii with medical imaging. Abdom Imaging.

[15] Outwater EK, Siegelman ES, Hunt JL (2001). Ovarian teratomas: tumor types and imaging characteristics. Radiographics.

[16] Matsuki M, Kaji Y, Matsuo M, Kobashi Y (2000). Struma ovarii: MRI findings. Br J Radiol.

[17] Qureshi M, Derebew L, Boucai L, Kishore P (2021). Retrospective diagnosis of malignant struma ovarii after discovery of pulmonary metastasis. AACE Clin Case Rep.

[18] Jung SE, Lee JM, Rha SE, Byun JY, Jung JI, Hahn ST (2002). CT and MR imaging of ovarian tumors with emphasis on differential diagnosis. Radiographics.

[19] Okada S, Ohaki Y, Inoue K, Kawamura T, Hayashi T, Kato T, Kumazaki T (2005). Calcifications in mucinous and serous cystic ovarian tumors. J Nippon Med Sch.

